# Extirpation of the Lachrymal Gland for the Cure of Fistula Lachrymalis

**Published:** 1845-04

**Authors:** Paul Bernard


*Extirpation of the Lachrymal Gland for the cure of Fistula Lachrymalis.* By M. Paul Bernard.—Some time ago M. Bernard communicated to the Academy of Sciences a case of cure of *fistula lachrymalis* by means of the extirpation of the lachrymal gland. This gland had only been previously extirpated on account of cancer; and the simplicity of the operation, as well as the rapidity of the cure, induced him to remove it for fistula. A man, 30 years of age, subject to a considerable discharge of tears from the left eye, but without fistula, had been subjected to most of the usual remedies for the cure of that disease. The canula, collyria, and ointments of various kinds, had been employed for a period of ten years, but the watering of the eye still continued, and rendered the vision very confused. M. Bernard then determined to remove at least that part of the gland which appeared to be hypertrophied. A vertical fold of the skin on the outer edge of the eye was raised, and a bistoury pushed through it. This exposed the palpebral edge of the gland. This portion was found to be hypertrophied; it was drawn out by the forceps, and removed. The orbital portion of the gland was not at this time removed; but as, a couple of months after, the watering of the eye still continued, he removed the orbital portion of the gland also. No bad results followed: the wound healed rapidly; the watering of the eye disappeared; in fact, a perfect cure followed.—*Ibid, from Rev. Med.*

